# A mixed-methods examination of clinicians’ perceived barriers to telehealth delivered applied behavior analysis

**DOI:** 10.3389/fpsyg.2023.1173644

**Published:** 2023-07-20

**Authors:** Anamiguel Pomales-Ramos, Hannah Tokish, Mya Howard, Diondra Straiton, Brooke Ingersoll

**Affiliations:** Michigan State Autism Lab, Psychology Department, College of Social Science, Michigan State University, East Lansing, MI, United States

**Keywords:** telehealth, barriers, applied behavior analysis, autism spectrum disorder, mixed methods, implementation outcomes

## Abstract

Following the COVID-19 pandemic, clinicians relied on telehealth to ensure continuity of essential healthcare services, such as Applied Behavior Analysis (ABA). Identifying barriers and examining them in the context of other implementation outcomes is important to support appropriate adaptations and sustainability of telehealth-delivered ABA services. Convergent mixed methods design was utilized to identify barriers experienced by ABA clinicians (*N* = 388) when delivering ABA services over telehealth to autistic children and their families following the first six months of the COVID-19 pandemic. Additionally, barriers were examined in relation to telehealth implementation outcomes and intentions for continued adoption. Findings reveal that clinicians rated providing direct services (*M* = 3.52, SD = 1.14) as more difficult than conducting assessments (*M* = 3.29, SD = 1.06), and both as more difficult than providing parent-mediated interventions [(*M* = 2.47, SD = 1.11), *F*(2, 381) = 162.26, *p* < 0.001]. A principal components analysis indicated a 3-factor solution of barriers related to: (1) technology (α = 0.82), (2) administrative tasks (α = 0.88), and (3) client characteristics (α = 0.88). The most frequently endorsed barriers were related to client characteristics, including increased difficulty providing telehealth services to children who elope (*M* = 4.37, SD = 0.81), children who exhibit challenging behaviors (*M* = 4.31; SD = 0.83), and children who are in the preverbal stage or use nonverbal language to communicate (*M* = 4.07; SD = 1.00). Fewer barriers related to client characteristics uniquely predicted implementation variables including acceptability, appropriateness, and feasibility. Thematic analysis revealed challenges related to technology, caregiver involvement, child engagement, implementation of intervention strategies over telehealth, and administrative or logistical barriers. These findings highlight the need for targeted strategies that facilitate telehealth use to address specific client needs and support the implementation of telehealth services in usual care settings.

## Introduction

Autism spectrum disorder (ASD) is characterized by difficulties with social communication as well as the presence of restricted and repetitive behaviors, interests, and activities (American Psychiatric Association, 2013). Despite a higher reported need for specialized, long-term healthcare services, autistic children and their families often face challenges in accessing essential assessment and intervention services. Barriers such as provider shortages, long waitlists, distance to specialized services, and costs can result in significant unmet healthcare service needs (Arim et al., 2017).

Telehealth can be leveraged to enhance access to mental health services and mitigate the effects of service disruptions. This service modality has been shown to deliver several benefits to mental healthcare systems, including alleviating staff shortages, lowering service costs, and boosting enrollment capacity and frequency of services (Sutherland et al., 2019; Rooks-Ellis et al., 2020; Camden and Silva, 2021).

One of the most widely used interventions for children on the autism spectrum in clinical settings is Applied Behavior Analysis (ABA). ABA is a type of early intervention based on behavioral learning principles that involves frequent instructional sessions to help children develop social communication, adaptive, play, and academic skills while reducing challenging behaviors. During ABA sessions, providers positively reinforce progress toward skill development goals and may analyze the communicative purpose of challenging behaviors to replace them with more adaptive behaviors that accomplish the same goal (Foxx, 2008).

Current literature on telehealth-delivered ABA services has demonstrated successful implementation of various techniques including functional analysis, functional communication training, naturalistic teaching, and parent-mediated interventions or parent training or caregiver coaching (Barretto et al., 2006; Lindgren et al., 2016; Tomlinson et al., 2018; Ferguson et al., 2019). Studies also suggest comparable improvement in behavior challenges and social communication skills for children with autism, in addition to increased parent knowledge and implementation of ABA techniques at home, across in person and telehealth-delivered ABA services (Peterson et al., 2017; Romani and Schieltz, 2017; Tomlinson et al., 2018; Blackman et al., 2020; Lindgren et al., 2020; Schieltz and Wacker, 2020; Unholz-Bowden et al., 2020; Pollard et al., 2021; Batton et al., 2022; Ferguson et al., 2022). Caregiver coaching *via* telehealth in ABA principles has led to high accuracy of caregiver implementation of intervention techniques with their child, and parents report satisfaction with and acceptability of telehealth delivery methods (Romani and Schieltz, 2017; Araiba and Čolić, 2022; Ferguson et al., 2022; Gerow et al., 2022). Other benefits of telehealth-delivered ABA have included cost savings, reduced travel burden, remote training of behavior analysts, and increased access to care (Lindgren et al., 2016; Peterson et al., 2017; Tomlinson et al., 2018; Ferguson et al., 2022).

While most of the evidence-base has built on the previous efficacy trials of ABA telehealth strategies, the increased use of telehealth services following the COVID-19 pandemic has shed light on the need for further exploration and understanding of its community-based implementation. As providers gain experience using this service modality in clinical or usual care settings, further research is needed to identify challenges to utilizing ABA telehealth services in order to support the use of best practices in real-world settings.

Much of the literature examining barriers to telehealth-delivered ABA services has been predominantly conducted in the context of caregiver coaching strategies. When coaching caregivers on ABA strategies over telehealth, clinicians have reported challenges with technical difficulties, cultural and language barriers, access to technology for underserved families, and transfer of specific ABA practices to telehealth (Lee et al., 2015; Tomlinson et al., 2018). Recently, Dueñas and D'Agostino (2022) were the first to expand on these findings by examining clinicians’ experiences delivering both direct (i.e., working directly with the child or adolescent over telehealth) and indirect (i.e., caregiver coaching) ABA telehealth services. The study employed qualitative methodology to explore ABA clinicians’ responses to open-ended questions related to barriers and facilitators experienced when delivering telehealth in clinical settings. Their findings highlighted challenges in transferring in-person ABA strategies to the telehealth modality, accessing technology and technological support, engaging with clients, and coaching caregivers who lack proficiency in facilitating services.

In order to build on these findings and gain a better understanding of how ABA providers in clinical settings are utilizing telehealth services, it is important to consider how these challenges influence other implementation outcomes, such as clinician perceptions of the acceptability, feasibility, and appropriateness of telehealth delivery of ABA services. Examining barriers in the context of implementation outcomes is important to develop appropriate telehealth adaptations of ABA and ensuring the scalability and sustainability of telehealth-delivered ABA programming (Tabak et al., 2012).

The current study utilizes a convergent mixed methods design to identify barriers to using telehealth to deliver ABA services to autistic children and their families following the first six months of the COVID-19 pandemic. The study expands on recent work by Dueñas and D'Agostino (2022) by using both quantitative and qualitative methodology to identify barriers, examining provider characteristics that influence their experiences with telehealth, and evaluating telehealth implementation outcomes in the context of ABA service provision (Proctor et al., 2011). Therefore, the current study has two main objectives:

1. Identify barriers and gain a comprehensive understanding of ABA clinicians’ unique challenges with this service modality.
2. Consider how barriers influence telehealth implementation outcomes and intentions for continued adoption of this service modality.

## Materials and methods

### Methodological and theoretical approach

Clinicians completed an online Qualtrics survey distributed nationally through the Behavior Analyst Certification Board between July and September of 2020 following IRB approval. The survey included closed- and open-ended survey items related to challenges experienced when delivering ABA services *via* telehealth to autistic children and their families. Items and measures included in the survey are described in the measures section below. A convergent mixed methods design (QUAN + qual) was used to identify perceived barriers to delivering ABA to autistic children and their families using telehealth and gain a comprehensive understanding of clinicians’ experiences with this modality (Palinkas et al., 2011). Quantitative methods (QUAN) were the prioritized method which provided information about the relationship between common barriers and implementation outcomes, while qualitative methods (qual) were the secondary method which provided deeper insight into unique challenges experienced by ABA providers. Quantitative and qualitative findings were integrated through the use of joint display tables that highlight key barriers and themes identified by the team.

### Participants

A total of 479 clinicians completed the online survey (Table 1). In order to participate, clinicians needed to be over the age of 18, certified through the Behavior Analyst Certification Board, and have at least one client diagnosed with autism spectrum disorder under the age of 21 in their caseload. The survey included branching logic to screen out providers who were ineligible. Of the total sample, 18.90% had experience providing telehealth services before social distancing guidelines in March 2020, 58.60% first provided telehealth services during the COVID-19 pandemic, and 22.50% had never provided telehealth services. The current study only examined participants who had experience providing telehealth services (N = 388). Participants resided in the United States (across 40 states) and predominantly identified as White (78.90%), non-Hispanic (76.80%), women (88.90%). Participants were certified as Board Certified Behavior Analysts (BCBA; Master’s level degree, 59.50%; Doctoral level degree, 3.60%), Board Certified Assistant Behavior Analysts (BCaBA; 2.80%), or Registered Behavior Technicians (RBT; 33.80%). They primarily provided ABA-telehealth services through an ABA-agency (70.40%), school (16.20%), private practice (11.10%), and community mental health clinic (6.40%).

**Table 1:** Provider demographic information (*N* = 388).

	*N*	%
Age (years), mean (SD)*	34.4 (9.4)	
Gender		
Cis-Male	41	10.6%
Cis-Female	345	88.9%
Trans	1	0.3%
Prefer not to answer	1	0.3%
Race		
American Indian/Alaska Native	10	2.6%
Asian	30	7.7%
Black/African American	27	7.0%
Native Hawaiian/Other Pacific Islander	7	1.8%
White	306	78.9%
Prefer not to answer	18	4.6%
Ethnicity		
Hispanic/Latinx	61	15.7%
Not Hispanic/Latinx	298	76.8%
Prefer not to answer	20	5.2%
Missing	9	2.3%
Highest education		
High School Graduate or GED	1	0.3%
Some college or specialized training	13	3.4%
Associate degree	7	1.8%
Bachelor’s degree	77	19.8%
Master’s degree	263	67.8%
Doctoral degree	27	7.0%
Certification		
Registered behavior technician (RBT)	131	33.8%
Board certified assistant behavior analyst (BCaBA)	11	2.8%
Board certified behavior analyst (BCBA)	232	59.5%
Board certified behavior analyst doctoral (BCBAD)	14	3.6%
Provides supervision		
Yes	241	62.1%
No	147	37.9%
Disciplinary background		
Applied behavior analysis (ABA)	333	85.8%
Social work	22	5.7%
Psychology	98	25.3%
Speech-language pathologist	8	2.1%
General education	34	8.8%
Special Education	121	31.2%
Early childhood education	47	12.1%
Occupational therapy	1	0.3%
Employment setting		
Comunity mental health center	25	6.4%
Applied behavior analysis (ABA) Agency	273	70.4%
Private practice	43	11.1%
School	63	16.2%
Specialty center	22	5.7%

*N = 383.

### Measures

#### Provider demographics

Clinicians’ demographic and professional characteristics were measured. Demographic characteristics included age, race and ethnicity, state of residence, and gender. Professional characteristics included certification, educational background, and highest degree earned.

#### Telehealth service delivery

In order to characterize telehealth service provision, participants completed multiple selection items (i.e., select all that apply) to report on service modality (videoconferencing, hybrid, and video recordings) and types of services (direct intervention, parent training, and assessment). When reporting on types of services, clinicians also rated the difficulty for each type service by using a 5-point Likert-scale (1-Extremely Easy; 5-Extremely Difficult). They also reported on training experiences they received to prepare for telehealth service delivery by completing a multiple selection item (i.e., select all that apply; Workshops/Webinars, Supervision, Observation, Self-Guided, and No training).

#### Penetration of telehealth services

In order to examine the extent to which telehealth was integrated within the practice setting (i.e., penetration; Proctor et al., 2011), clinicians completed three questions related to their caseloads before COVID-19, the number of clients who were offered telehealth services, and clients who followed through with attending at least one session over telehealth.

#### Barriers to telehealth services

An *a priori* list of barriers to delivering telehealth services was developed from a comprehensive literature review examining the use of telehealth to deliver broader mental health services (Gros et al., 2013; Lee et al., 2015; Ashburner et al., 2016; Lindgren et al., 2016; Peterson et al., 2017; Tomlinson et al., 2018; Campbell et al., 2019; Hoffmann et al., 2019). Items were primarily related to technology, client, personal, and organizational challenges. Each item was then tailored to the experiences of autistic children and families (refer to Tables 2–4 quantitative data section for full list of items). Clinicians rated each barrier using a 5-point Likert-scale (1-Extremely Easy; 5-Extremely Difficult). Three scales were developed from the list of items using a principal components analysis, as described in the results section. Additionally, in order to understand unique barriers experienced when using telehealth to deliver ABA services to autistic children and their families, clinicians were asked to complete an open-ended survey question (“What challenges or barriers did you experience when providing ABA services over telehealth?”). Responses could be typed into a textbox without a word limit.

**Table 2:** Joint display for quantitative and qualitative data related to technological barriers.

Quantitative data (*N* = 388)		
Technological barriers scale (α = 0.82)	*M(SD)*	*%*
Client’s access to technology (e.g., computer, tablet, smart phone)	2.99 (1.31)	47.20%
Client’s access to internet services	2.74 (1.35)	36.10%
Client’s familiarity with technology or videoconferencing software	3.22 (1.19)	10.60%
Clinician’s access to an encrypted computer, tablet, or smart phone	1.97 (1.19)	13.90%
Clinician’s access to internet services	1.63 (0.99)	7.50%
Clinician’s access to HIPAA compliant videoconferencing software	2.11 (1.21)	18.10%
Clinician comfort with technology or videoconferencing software	1.94 (1.07)	11.60%
Internet connectivity issues	2.83 (1.23)	37.60%

**Qualitative data (*N* = 100)**			
*Technological barriers theme*	*n*	*Representative quotes*	*Interpretation*
Internet connectivity issues	18	*“Technology issues, difficult to hear both ways and limited view of the environment.”*	Clinicians identified issues with equitable access to technology, as well as connectivity issues that prevented them from clearly seeing or hearing the client.
Difficulty seeing or hearing child	18		
Access to technology	10		

**Table 3:** Joint display for quantitative and qualitative data related to client barriers.

**Quantitative data (*N* = 388)**		
*Client barriers scale (α = 0.87)*	*M (SD)*	*%*
Provide telehealth to clients who elope or bolt.	4.37 (0.81)	86.90%
Provide telehealth to clients who engage in challenging behaviors.	4.31 (0.83)	83.50%
Provide telehealth to clients in preverbal stage.	4.07 (1.00)	76.80%
Provide telehealth to clients who are in early stages of services	3.9 (0.99)	74.50%
Provide telehealth to clients who experience motor difficulties.	3.67 (0.86)	59.50%
Provide telehealth to clients who communicate using single words.	3.64 (1.04)	60.30%
Transition clients from in-person to telehealth services.	3.53 (1.01)	62.70%
Have a productive session over telehealth.	2.98 (1.13)	39.90%
Make session feel personal	2.91 (1.22)	40.20%
Establish rapport over telehealth.	2.8 (1.13)	32.80%

**Qualitative data (*N* = 100)**			
*Caregiver involvement theme*	*n*	*Representative quotes*	*Interpretation*
Distractions and competing demands	17	*“If families had multiple kids at home it was hard to do therapy. If the parents did not want to have to help during the session, they denied wanting therapy.”*	Clinicians noted increased caregiver participation in sessions. Some clinicians had difficulties navigating caregivers’ perceptions of telehealth services and competing demands that limited their involvement in sessions.
Expectations and perceptions of services	16		

*Child Engagement theme*	*n*	*Representative quotes*	*Interpretation*
Difficulty maintaining attention	54	“There were challenges in keeping client engaged during telehealth, and during behavior modifications for client to respond.”	Clinicians described the influence of child characteristics on the ease of telehealth services, with some struggling to address or manage certain behaviors or presentations.
Elope from session	12		
Age or language level	15		

*Implementing intervention strategies theme*	*n*	*Representative quotes*	*Interpretation*
Behavioral management skills	27	“Intervening to address maladaptive behaviors was often a challenge and certain skills could not be practiced at all.”	Clinicians struggled to implement intervention strategies over telehealth, including behavioral management, prompts, and reinforcements. Some reported relying on caregivers during sessions, however experienced difficulties coaching caregivers or demonstrating strategies over telehealth.
Demonstrating strategies or coaching parents	19		
Prompting or reinforcing the child	21		

**Table 4:** Joint display for quantitative and qualitative data related to administrative barriers.

Quantitative data (*N* = 388)		
*Administrative barriers scale (α = 0.88)*	*M (SD)*	*%*
Access to research about telehealth services	3.07 (1.13)	40.40%
Completing intakes or on-boarding new clients	3.17 (1.04)	37.70%
Access to therapy or assessment materials (e.g., manuals)	2.79 (1.19)	35.50%
Remote record keeping and paperwork	2.51 (1.18)	26.30%
Privacy and confidentiality	2.55 (1.18)	25.00%
Establishing professional boundaries and self-disclosure	2.54 (1.16)	24.50%
Scheduling appointments with my clients	2.42 (1.17)	24.30%
Using an interpreter	3.18 (0.89)	23.50%
Cost of systems (e.g., software, technology)	2.76 (1.05)	23.40%
Providing families with materials (e.g., worksheets)	2.74 (1.18)	19.60%
Providing assessment reports	2.66 (0.98)	19.30%
Providing remote consenting for telehealth services	2.47 (1.05)	18.30%
Concerns about mandated reporting	2.57 (1.05)	15.70%
Reimbursement from insurance	2.76 (0.92)	14.70%
Liability insurance coverage for specific services provided	2.55 (1.01)	12.10%

**Qualitative data (*N* = 100)**			
Administrative or logistical barriers theme	*n*	Representative quotes	Interpretation
Administrative barriers related to lack of guidance	7	*“Yes, clarity on if doing the proper programs over telehealth.”*	Overall, clinicians felt unprepared to deliver telehealth services due to lack of guidance or information on how to implement services using this modality and lack of resources to support sessions.
Lack of resources	6	“Having to create materials for parents to study and implement.”	

#### Implementation variables

Acceptability, appropriateness, and feasibility were measured using various 4-item measures that used a 5-point Likert scale (1-Completely Disagree; 5-Completely Agree). For each scale the items were averaged for an overall rating. The Acceptability of Implementation Measure was used to assess the extent to which ABA clinicians perceive telehealth service modality as agreeable, palatable, or satisfactory (AIM; Weiner et al., 2017). The Intervention Appropriateness Measure was used to assess the extent to which clinicians perceive telehealth service modality as a good fit, relevant, and compatible with ABA services (IAM; Weiner et al., 2017). The Feasibility of Implementation Measure was used to assess clinicians’ perception of the extent to which telehealth can be successfully used to deliver ABA services (FIM; Weiner et al., 2017). Cronbach’s alpha indicated excellent internal consistency for the AIM (α = 0.95), IAM (α = 0.95), and FIM (α = 0.91).

### Data analysis

#### Quantitative data analysis

Descriptive statistics were used to examine clinicians’ telehealth training experiences and penetration of telehealth services during the COVID-19 pandemic. Telehealth penetration was calculated by using the number of families who were offered telehealth session as a numerator and the number of families in the caseload as a denominator, as well as the number of families who attended a telehealth session as a numerator and the number of families who were offered telehealth session as a denominator. A repeated measures ANOVA was used to compare clinicians’ difficulty implementing specific services over telehealth (i.e., assessments, direct interventions with clients, and parent/caregiver training). Individual barriers were examined using descriptive statistics in order to identify the most frequently endorsed barriers. A principal components analysis (PCA) with varimax rotated factors and Kaiser Normalization was conducted with barrier items to identify meaningful subscales. Cronbach’s alphas were calculated on the factors suggested by the PCA in order to examine the internal consistency of the factors. A repeated measures ANOVA was used to compare clinicians’ ratings for the barriers scales. Hierarchical linear regressions were used to examine predictors of telehealth implementation outcomes (i.e., acceptability, feasibility, and appropriateness; Proctor et al., 2011) and inform on potential strategies to facilitate the implementation of telehealth services. Provider demographics associated with implementation outcomes were entered into the first step and barriers subscales were entered into the second step. A Pearson’s correlation was used to examine implementation outcomes (using the average overall score for each implementation outcome) as predictors for intentions for continued telehealth use.

#### Qualitative analysis

A team of three coders used thematic analysis to examine the responses to the open-ended question survey question about challenges experienced while delivering ABA services over telehealth (Clarke et al., 2015). An inductive approach was used to identify major codes and patterns in the data. During the first phase of coding, the team became familiar with the data and independently generated a list of codes. Then, the team discussed and refined the codes through multiple meetings, resulting in 8 codes. The responses were consensus-coded using the finalized list of codes on Dedoose. After examining the responses with the assigned codes, the team organized and consolidated codes through an iterative process in order to identify major themes and subthemes that represented the patterns in the data. All team members reached agreement on the final codes assigned to each response. Finally, theme frequency was calculated in order to converge the data with the qualitative findings. Frequencies included the percentage of providers who commented on a theme, as well as the total number of comments that were related to each subtheme. Data integration of quantitative and qualitative findings involved examining which qualitative themes provided robust information and elaborated upon the quantitative ratings of common barriers. Joint display tables were used to integrate quantitative data and qualitative themes and a narrative discussion in the “discussion” section was used to further elaborate on unique barriers identified.

## Results

### Telehealth service delivery

At the time of the survey, 86% of clinicians reported their clinic or organization was providing in-person services. Of their total caseload, they offered telehealth services to 40% of their clients. Of those clients, 92.28% attended at least one session over telehealth.

In terms of telehealth service provision, 84.63% of clinicians provided direct intervention with clients, 56.87% provided parent/caregiver training or caregiver-mediated services, and 37.46% conducted assessments. A repeated measures ANOVA revealed clinicians rated providing direct services (*M* = 3.52, SD = 1.14) as more difficult than conducting assessments (*M* = 3.29, SD = 1.06), and both as more difficult than providing parent mediated interventions [(*M* = 2.47, SD = 1.11), *F*(2, 381) = 162.26, *p* < 0.001]. When providing parent-mediated interventions, about 72% of clinicians reported using a parent-mediated program of their own design (i.e., providers select strategies and coach the parent on strategies the clinician would otherwise be implementing directly with the child during an in-person session) and 28% used a manualized parent-mediated evidence-based program (i.e., a program designed to teach parents strategies for which researchers have provided acceptable level of research showing positive outcomes for children and their families). When providing telehealth sessions to children and families, clinicians predominantly used live videoconferencing to deliver services (86.83%), combined both in-person and telehealth services (47.73%; hybrid), or asked caregivers to send video recording of their child for evaluation or feedback (8.23%).

To prepare for telehealth service delivery, 25% of clinicians attended a telehealth workshop or webinar, 23.5% received supervision by another clinician, 22.4% observed another clinician delivering services over telehealth prior to their first case, 38.1% engaged in self-guided learning by independently reading empirical papers and looking for resources online, and 37.4% did not receive any training.

### Barriers to telehealth service provision

#### Quantitative analysis

The most frequently endorsed barriers were related to client characteristics, including increased difficulty providing telehealth services to children who elope (*M* = 4.37, SD = 0.81), children who exhibit challenging behaviors (*M* = 4.31; SD = 0.83), and children who are in the preverbal stage or use nonverbal language to communicate (*M* = 4.07; SD = 1.00). Clinicians also frequently endorsed challenges with providing telehealth services to children and families who are in the early stages of services (*M* = 3.9, SD = 0.99), as well as transitioning clients from in-person services to telehealth services (*M* = 3.53, SD = 1.01).

The principal components analysis with varimax rotation was used to identify meaningful subscales. A three-factor solution was selected based on examination of the eigenvalues (>1), scree plot, and interpretability. The first three factors accounted for 40.65% of the variance. Factors were related to difficulties with technology, administrative and logistical tasks, and targeting specific client characteristics. Thus, three barriers scales were developed: the technological barrier scale was comprised of 8 items (Table 2), the administrative scale was comprised of 15 items (Table 4), and the client barrier scale was comprised of 10 items (Table 3). Cronbach’s alpha indicated good internal consistency for all factors [Technology (α = 0.82); Administrative tasks (α = 0.88), and Client characteristics (α = 0.87)]. A repeated measures ANOVA examining the three barriers scales revealed clinicians rated client characteristics barriers (*M* = 3.61, SD = 0.69) as significantly more difficult than administrative barriers (*M* = 2.77, SD = 0.65), and both as significantly more difficult than technological barriers [(*M* = 2.42, SD = 0.75), *F*(2, 381) = 627.25, *p* < 0.001].

#### Qualitative analysis

A total of five themes emerged regarding perceived barriers to ABA telehealth service provision: (1) technological barriers, (2) challenges with caregiver involvement, (3) difficulties with child engagement, (4) difficulties implementing intervention strategies over telehealth, and (5) administrative or logistical barriers.

#### Technological barriers

Challenges related to technology were endorsed by 84% of the clinicians. These difficulties were primarily related to connectivity issues (*n* = 18), challenges seeing or hearing the clients (n = 18), and disparities in access to technology and internet (*n* = 10).

Clinicians described either personally experiencing connectivity issues or their clients experiencing connectivity issues that interrupted the session. Some clinicians also noted that these issues would influence their ability to hear or see the client, resulting in missing context or important information during sessions.

*“Yes, we have been dealing with tech issues for years. Primarily the inability to see the entire environment and to hear when the video/audio freezes.”*

Additionally, the camera placement, lag times, or the type of device used (i.e., smart phones) sometimes limited their field of view and clinicians were not able to assess the environment or see all responses if the child or caregiver moved and faced away from the camera:

*“There were limitations of field of view or I was limited to only visual and auditory stimuli. There was difficulty observing stimuli evoking client response. Connectivity issues and slight lag time also affected this.”*

*“When parents used cell phones,* versus *tablets or laptops, it was a challenge because I could not see as well.”*

Clinicians also felt they did not have access to the technological equipment and resources needed to provide telehealth services. For example, one clinician noted:

*“Yes internet problems, company did not provide laptops or pay for internet or equipment such as headphones.”*

They also indicated challenges with access to resources and equipment needed to provide or receive telehealth services. They noted families from specific counties or low-income neighborhoods may have difficulties accessing Wi-Fi or equipment needed to engage in telehealth services:

*“Low income families often lacked wifi or internet.”*

*“We also had frequent tech issues. Some kids live in the county where the Internet is not as good.”*

#### Challenges with caregiver involvement

A total of 45% of clinicians reported difficulties with caregiver involvement in telehealth services, such as increased distractions and competing demands (*n* = 17) and navigating conversations around caregivers’ expectations and perceptions of telehealth services (*n* = 17).

Clinicians described that when implementing ABA services over telehealth, caregivers play a major role in the successful delivery of services. In order to more efficiently deliver services, clinicians often relied on caregivers being involved and present in session to implement services and help keep the child engaged.

*“Parent involvement played a huge role in how successful the client was receiving services.”*

*“I found an increase in parent involvement which was great!!!!”*

*“Many parents struggled to attend to all directions given, while others did well and ended up learning a lot about their child’s program.”*

However, clinicians frequently described challenges with delivering services when caregivers were unable to attend or engage in session due to other responsibilities or stressors, such as caretaking of other children or family members and work demands. It is also important to note that the majority of clinicians who participated in this survey were delivering telehealth services in the context of COVID-19 pandemic, a period in which there was an increase in caregiver demands and stressors.

*“Hard to provide services when multiple children have services with a single parent.”*

*“Some parents [were] unable to attend or engage during sessions due to other scheduling, multiple kids in home, or just unwillingness to participate in session.”*

*“Some difficulty with certain behavioral interventions due to number of kids in the home or other responsibilities (parents working from home).”*

*“Not all parents had time to be full participants in the process.”*

With increased involvement in session, clinicians described that sometimes this resulted in negative perception of services. Some parents struggled with directly implementing strategies, which led to increased frustration. Additionally, other parents were frustrated with the pace of sessions due to additional time spent with parent coaching, technological issues, or changes in goals to fit the service modality.

*“If the parents did not want to have to help during the session, they denied wanting telehealth therapy.”*

*“Parents seemed frustrated that the implementation is slower than anticipated.”*

#### Challenges with child engagement

Barriers related to difficulty engaging clients were endorsed by 61% of the clinicians. More specifically, clinicians reported challenges regarding maintaining the child’s attention during sessions (*n* = 45) and preventing the child from leaving or eloping from the session (*n* = 12). For example, a clinician noted:

*“Harder to manage off-task behavior of clients when you are not physically present.”*

Clinicians described that technology and the equipment needed to conduct telehealth sessions was often a distraction for children. Children became easily distracted with buttons or other online activities.

*“Clients had difficulty refraining from touching computer buttons throughout session, leading to calls being ended or being distracted by pushing buttons repeatedly.”*

*“The child would sometimes swipe on the device and lose connection to an extent,* etc. *It was hard to teach the parent how to prevent those kinds of things.”*

In order to help with managing distractions, clinicians implemented strategies such as training parents on behavioral strategies to mediate distractions, asking parents to sit next to the child to provide additional supervision, and providing frequent breaks. However, some noted that despite implementing supports to help with attention, they continued to struggle to maintain the child’s engagement. Others noted that attempting to re-engage clients and implementing these strategies was time consuming, changed the structure of the sessions, and limited their ability to focus on other goals.

*“Yes. It was difficult for some clients to attend to the telehealth session even if the parents were sitting next to them.”*

*“The child was not able to sit by self during service, so mom or dad take turns which limits the length and amount of session.”*

*“Challenges with clients’ tolerance in front of computer. Frequent breaks allotted.”*

*“When client attempts to elope it is difficult to help them focus and calm down.”*

Clinicians also indicated increased difficulty implementing intervention strategies over telehealth depending on specific client characteristics, such as age and verbal language level. They noted younger children were more difficult to engage over telehealth, as well as children who demonstrate more limited verbal communication skills.

*“With younger children with behavioral challenges, it’s tricky to keep them engaged over a screen.”*

*“The most challenges came from working with clients who are almost nonverbal and have difficulty staying engaged during virtual services.”*

*“Yes - not recommended for all my clients/students. The higher verbal ability the easier it was to provide support. Those who eloped were much harder [for] telehealth [to] work with as well.”*

#### Implementing intervention strategies over telehealth

A total of 57% of clinicians reported experiencing challenges when trying to implement intervention strategies over telehealth. More specifically, they frequently described barriers related to implementing behavioral management skills (*n* = 27), demonstrating strategies or coaching parents (*n* = 19), and prompting or reinforcing the child (*n* = 21).

With increased caregiver involvement in sessions, clinicians had to engage in more parent coaching strategies over telehealth, such as describing strategies. Some clinicians indicated they struggled to demonstrate techniques or coach parents to learn new techniques and implement corrective feedback:

*“Trying to explain to parents interventions without being able to demonstrate in person.”*

*“It was difficult to guide the parents on the spot in how to prompt or redirect the child.”*

*“Some challenges were the parents being receptive to corrective feedback, as well as implementing the feedback given.”*

They also noted concerns with the quality or fidelity of the intervention as the parent implemented it with the child. For instance, one clinician noted:

*“Parent involvement is unpredictable and may compromise the effectiveness of prompting or error correction.”*

Clinicians also described challenges when their client engaged in challenging behaviors or aggressive behaviors during the session. They noted difficulties with directly implementing behavioral management skills with the child over telehealth:

*“Intervening to address maladaptive behaviors was often a challenge and certain skills could not be practiced at all.*

*“When maladaptive behaviors occur there is minimal we can do through the screen.”*

Other clinicians relied on parents to implement behavioral management skills and provided live coaching and feedback on how to respond to the child. However, some described challenges with coaching parents on behavioral management skills:

*“Yes there were challenges, it’s difficult to coach parents to address aggression via Telehealth.”*

*“It is hard to give in the moment feedback when challenging behavior is occurring.”*

Clinicians working directly with clients over telehealth commented on challenges related to providing prompting and reinforcements. For example, telehealth services limited the use of physical prompting during sessions:

*“Unable to provide prompting involving proximity, hand-over-hand.”*

They also noted differences in responses to reinforcements over telehealth. For example, clinicians struggled to provide more naturalistic reinforcements when working directly with the child over telehealth.

*“Providing social and natural reinforcement is much more difficult.”*

Another clinician described their clients as indifferent to praise when delivered over telehealth:

*“Also, the praise delivered does not seem as appealing/desired by client.”*

A clinician explained that changes in their ability to consistently reinforce the child over telehealth resulted in regressions in previously observed treatment gains:

*“Higher rates of escape maintained behavior due to intermittent reinforcement (cannot physically follow through without parent training on how), shorter session due to attention span of client therefore leading to more intrusive reinforcement strategies that were previously faded,* etc.*”*

#### Administrative or logistical barriers

A total of 14% of clinicians reported administrative or logistical barriers. Clinicians described administrative barriers related to lack of guidance or information (*n* = 7) and lack of resources (*n* = 6).

Clinicians noted feeling unclear on what programs and strategies to implement over telehealth and lacking guidance on how to provide telehealth services. They also noted lacking resources to train other clinicians to provide telehealth services.

*“Yes, clarity on if doing the proper programs over telehealth.”*

*“So many! Staff training in new methodologies and lack of resources.”*

Other clinicians described challenges with the initial stages of telehealth services while they adjusted to the new modality and learned what worked best for families:

*“The initial barrier was adjusting to telehealth and learning what works for families.”*

When implementing traditional services with children and families, clinicians often rely on therapy materials to support learning (such as worksheets, visual schedules, pictured items, toys that enhance learning). When they transitioned to telehealth services, clinicians experienced barriers with accessing and creating new therapy materials that were compatible with the telehealth modality (such as PDF forms, power point slides to provide visuals). Additionally, having to create and compile these resources was time consuming. For example, a clinician expressed:

*“Initially it was recommended to have the clients with a set of materials and I had the same set of materials and we worked on the video... this was very complex. While working with a parent, we discussed Powerpoint presentation and started providing the sessions with a powerpoint in early March. Making the powerpoints have been very time consuming.”*

It is also important to consider the context in which clinicians reported these barriers. During the COVID-19 pandemic, clinicians were faced with having to quickly transition services to a telehealth modality in order to avoid treatment disruptions. This rapid transition of services left clinicians with limited time to gather resources and supports to prepare families for a new type of service modality.

*“Also, our clinic abruptly shut down which did not give us enough time to prepare the patients or the families.”*

*“It was very difficult since our clients were not familiar with the model, their routines and home-life were completely altered, and it was a challenge for us to quickly develop a telehealth model.”*

#### Clinician reported implementation outcomes

Pearson’s correlations were used to examine clinician background variables: clinician age, clinician gender (cis-male or cis-female), BCBA certification (RBT/BCaBA or BCBA/BCBA-D), and clinician training experience that were significantly related to implementation variables (average overall score for acceptability, appropriateness, and feasibility measures). BCBA certification was significantly related to acceptability (*r* = 0.16, *p* = 0.003), feasibility (*r* = 0.11, *p* = 0.01), and appropriateness (*r* = 114, *p* = 0.04). Clinician training experience was also significantly related to acceptability (*r* = 0.13, *p* = 0.02), feasibility (*r* = 0.18, *p* = <0.001), and appropriateness (*r* = 0.14, *p* = 0.01). Neither clinician age nor gender were significantly correlated to any of the implementation variables.

Hierarchical linear regressions were used to examine predictors of implementation variables. Clinicians’ telehealth training experience and BCBA certification were added to the first step of the model, then technology-, client-, and administrative-barrier scales were introduced to the final step of the model.

#### Predictors of acceptability

A hierarchical linear regression examining clinician’s background and barriers subscales was significant [*F*(5, 327) = 38.53, *p* < 0.001] and accounted for 37.10% of the variance in clinicians’ perceived acceptability of telehealth services (Table 5). Variance in the final step of the model was uniquely explained by BCBA certification and fewer reported barriers related to client characteristics.

**Table 5:** Hierarchical linear regression examining factors predicting telehealth acceptability.

	Model 1		Model 2	
Variable	*β*	*t*	*β*	*t*
BCBA certification	0.16	2.95*	0.20	4.45**
Telehealth training experience	0.14	2.52*	0.05	1.04
Technological barriers scale			−0.04	−0.68
Client barriers scale			−0.12	−1.90
Administrative barriers scale			−0.48	−8.61**
*R*^2^	0.043		0.371	
*F* change	7.38**		56.79**	

**p < 0.001; *p < 0.01.

#### Predictors of appropriateness

A hierarchical linear regression examining clinician’s background and barriers subscales was significant [*F*(5, 326) = 32.03, *p* < 0.001] and accounted for 32.9% of the variance in clinicians’ perceived appropriateness of telehealth services (Table 6). BCBA certification and fewer reported barriers related to client characteristics accounted for the variance in the final step of the model.

**Table 6:** Hierarchical linear regression examining factors predicting telehealth appropriateness.

	Model 1		Model 2	
Variable	*β*	*t*	*β*	*t*
BCBA certification	0.110	2.034*	0.15	3.21**
Telehealth training experience	0.141	2.603*	0.05	1.19
Technological barriers SCALE			−0.05	−0.83
Client barriers scale			−0.12	−1.81
Administrative barriers scale			−0.45	−7.78**
*R*^2^	0.031		0.329	
*F* change	5.34*		48.29**	

**p < 0.001; *p < 0.01.

#### Predictors of feasibility

A hierarchical linear regression examining clinician’s background and barriers subscales was significant [*F*(5, 326) = 34.10, *p* < 0.001] and explained 34.30% of the variance in clinicians’ perceived feasibility of telehealth services (Table 7). Variance in the final step of the model was uniquely explained by BCBA certification, telehealth training experiences, and fewer reported barriers related to technology, administrative logistics, and client characteristics.

**Table 7:** Hierarchical linear regression examining factors predicting telehealth feasibility.

	Model 1		Model 2	
Variable	*β*	*t*	*β*	*t*
BCBA certification	0.13	2.44*	0.18	3.87**
Telehealth training experience	0.19	3.48**	0.10	2.30*
Technological barriers scale			−0.12	−2.11*
Client barriers scale			−0.15	−2.36*
Administrative barriers scale			−0.37	−6.48**
*R*^2^	0.051		0.343	
*F* change	8.84**		48.39**	

**p < 0.001; *p < 0.01.

#### Intention for future use

Approximately 64% of clinicians reported they intended to continue using telehealth services in the future and after COVID-19 social distancing restrictions are lifted. A Pearson’s correlation revealed that acceptability (*r* = 0.62, *p* < 0.001), feasibility (*r* = 0.51, *p* < 0.001), and appropriateness (*r* = 0.55, *p* < 0.001) were strongly related to clinicians’ intentions for future use of telehealth services.

## Discussion

The current study aimed to gain a comprehensive understanding of the challenges and barriers ABA-clinicians faced when delivering telehealth services to autistic children and their families. The integration of quantitative and qualitative findings suggest that clinicians experienced barriers related to supporting and addressing specific technology (Table 2), client characteristics (Table 3), and administrative logistics (Table 4).

Clinicians described the influence of child characteristics on ease of telehealth delivery, noting that it can be difficult to work with children exhibiting externalizing behaviors, who elope or bolt from session and are in the preverbal stage or have limited expressive language skills. Supporting these characteristics over telehealth have also been reported as challenging in previous studies examining telehealth delivered assessments and other behavioral interventions for autistic children (Campbell et al., 2019; Hao et al., 2021; Kryszak et al., 2022). Clinicians in the current study commented on struggling to manage the environment to effectively promote the child’s engagement and facilitate attention over telehealth, as well as struggling to deliver behavioral management strategies over telehealth. This suggests a need for targeted strategies that facilitate telehealth ABA services for these clients.

The type of service delivered over telehealth also influences clinicians’ perceptions of the ease of use of telehealth services. Clinicians reported predominantly providing ABA services directly to the child, as opposed to primarily mediated by a caregiver. Furthermore, quantitative findings reveal that providing direct ABA services was perceived as significantly more difficult than providing parent-mediated interventions over telehealth. This is consistent with literature examining telehealth-delivered assessments for autistic children, which indicate that while building rapport *via* telehealth with parents is less difficult than expected, it can be challenging to engage with children (Kryszak et al., 2022).

Despite intending to provide direct ABA services to the child over telehealth, clinicians described that they often relied on caregiver involvement to engage the child or implement strategies. The majority of clinicians noted that when delivering parent-mediated interventions, they utilized a program of their own design (i.e., in which they used their clinical judgment to select and teach strategies to parents), as opposed to an evidence-based manualized parent-mediated intervention (i.e., intervention with well-established research that systematically teaches parents how to implement strategies). Thus, this suggests that clinicians attempted adapting the strategies they typically administer directly to the child in an in-person session and relied on the caregiver for assistance or support as needed. This is consistent with previous literature stating that clinicians struggle to transfer in-person strategies to the telehealth modality (Rodriguez, 2020; Dueñas and D'Agostino, 2022). In order to transfer these strategies to telehealth, clinicians in the current study commented that they relied on caregivers to implement services and follow through with prompting. However, they noted difficulties implementing caregiver coaching strategies, such as describing strategies and providing feedback in real time. This was particularly difficult when the child needed behavioral management skills or required more supportive prompting. Overall, these findings are consistent with Dueñas and D'Agostino’s (2022), who indicated that lack of caregiver knowledge of intervention strategy and lack of experience implementing plans were barriers to delivering ABA telehealth services. Thus, taken together, these findings suggest that a potential facilitator for ABA telehealth services could be increased professional training experiences related to parent-mediated intervention strategies and evidence-based parent coaching programs.

The pandemic restrictions prompted many families to try telehealth services for the first time (White et al., 2021). Results from this study suggest that clinicians and families experience an adjustment period to telehealth services. Clinicians indicated difficulties providing services to new clients who are in the early stages of services as well as transitioning existing clients from an in-person to a telehealth service modality. They described challenges navigating differences in caregivers’ expectations and perceptions of telehealth services. While clinicians reported that the majority of families who were offered telehealth services attended at least one session, they also noted that some families declined services due to increased involvement and negative perception of telehealth services.

These results provide further evidence that support the recommendations highlighted in Dueñas and D'Agostino’s (2022) discussion, which commented on the need to assess barriers before transitioning to telehealth services and for the development of programs to prepare families and ease the transition to telehealth services. The findings in the current study indicate the need for providing increased support and establishing clear expectations during the initial stages of services to facilitate the transition to telehealth services and to engage families more readily. During the consultation period preceding services, clinicians should assess caregivers’ knowledge and comfort with intervention strategies in order to select what services can be implemented over telehealth. For example, incorporating a parent training program during the transition to and the early stage of telehealth services could support caregivers who are less familiar with ABA strategies, given clinicians’ observation of increased caregiver involvement in telehealth sessions.

Following the rapid transition to telehealth services during early stages of the pandemic, various preliminary guidelines were developed to support telehealth services (Colombo et al., 2020; Cox et al., 2020). For example, Lerman et al. (2020) developed a checklist based on their data on telehealth delivered ABA parent coaching that helps clinicians consult logistics with the clients prior to transitioning to the telehealth modality. Rodriguez (2020) developed a clinical decision guide to selecting types of ABA services (e.g., direct services, behavior management, parent coaching) in response to the urgent need for guidance during the pandemic. An important next step would be to use this information on experiences with both direct and parent coaching services to update guidelines and recommendations as well as to develop a measure to identify *a priori* which families may need additional support to be successful with telehealth.

The challenges associated with the transition to telehealth services were exacerbated by the COVID-19 pandemic, as clinicians were required to make a quick transition to this service modality to ensure continuity of care. Clinicians felt they did not have enough time to prepare for telehealth service provision. For example, they were not able to readily access evidence-based information about telehealth services (i.e., research studies) or materials and visuals for the virtual modality. Many clinicians in the current study reported not receiving any training on telehealth service provision. They described feeling they lacked guidance on how to select and implement programs over telehealth. Literature examining the effectiveness of ABA telehealth services during the pandemic, both in the United States and other countries, highlights the experiences reported by clinicians in the current study. Studies examining the transition to telehealth services in clinic settings in India and Italy have described similar barriers, noting that clinicians had to create their own protocols and materials ahead of each session during the initial stages of the pandemic due to the lack of online resources and telehealth specific guidelines available (Degli Espinosa et al., 2020; Awasthi et al., 2021). Access to evidence-based practices and materials may facilitate clinicians’ perceived self-efficacy when delivering telehealth services and should be studied further.

In terms of technological barriers, clinicians identified concerns related to access to technology for families in under-resourced communities. Individuals in under-resourced communities are less likely to have a computer or home broadband services (Pew Research Center, 2021). Instead, they are more likely to rely on smartphones for online services (Pew Research Center, 2021) which clinicians in the current study reported made it more difficult to hear or see the client. Assessing which technology or resources families have access to is important to determining how to best leverage their resources to support services and assess whether telehealth services are the right fit. While qualitative frequencies reveal that clinicians frequently commented on technology-related barriers, quantitative results indicated that these challenges were given lower ratings on average. This suggests that while clinicians often encounter technological barriers, they believe that technology issues could be overcome and do not interfere with telehealth outcomes (Gros et al., 2013; Ashburner et al., 2016; Hao et al., 2021; Southey and Stoddart, 2021; Spain et al., 2021).

This study also aimed to identify factors that influence implementation outcomes which are significantly related to intentions for continued use (Proctor et al., 2011). Findings show that BCBA certification, which requires additional training on ABA strategies, predicted increased acceptability and appropriateness of telehealth services. Participating in professional training experiences about the telehealth modality was predictive of increased perceived feasibility of delivering ABA services over telehealth. In terms of perceived barriers, clinicians who experienced fewer barriers related to client characteristics reported increased acceptability, feasibility, and appropriateness. Fewer reported barriers related to technology and administrative logistics predicted increased perceived feasibility. These findings, taken together with the barriers identified in this study, suggest that clinicians may benefit from receiving increased training on evidence-based parent-mediated interventions, particularly since greater difficulty in managing specific client needs may require increased caregiver involvement. Moreover, the development of professional training experiences related to telehealth service provision, specifically for the delivery of ABA strategies, may facilitate the implementation of telehealth services, as it provides clinicians with the tools to address their clients’ needs. Given that these implementation outcomes influence intentions for future use and that many clinicians reported they intend to continue using telehealth in the future, it is crucial to continue developing supports to facilitate the successful implementation of telehealth services.

### Limitations

The current study has limitations that should be considered when interpreting the results. The data presented in this study were collected during the COVID-19 pandemic. Clinicians’ experiences and perceptions of telehealth service provision was evaluated during a time of disruption and increased challenges. Additionally, the sample of ABA providers may not be fully representative of the community of ABA providers and their experiences, due to the variability in coverage and adoption of telehealth services among different states and health care funders. Also, the barriers scale was developed for this study and has not been validated. Additionally, a follow up survey would provide information on how clinician’s experiences with telehealth over time have influenced their perceptions about telehealth and barriers experienced. Finally, while intention to use telehealth in the future is a good predictor of actual use (Ajzen, 1991), longitudinal data and a follow up survey would be important to determine to what extent barriers and implementation outcomes are predictive of sustainability and future use.

## Data availability statement

The raw data supporting the conclusions of this article will be made available by the authors, without undue reservation.

## Ethics statement

The studies involving human participants were reviewed and approved by Michigan State University Human Research Protections Program. The patients/participants provided their written informed consent to participate in this study.

## Author contributions

AP-R, DS, and BI led the design of the study, gathered, and organized the data. AP-R and BI analyzed and interpreted the data. AP-R, MH, and HT wrote the manuscript with contributions from all authors. All authors contributed to the article and approved the submitted version.

## Funding

AP-R and DS funded the publication of this manuscript with support from the Michigan State University’s College of Social Science COVID-19 small research grant.

## Conflict of interest

The authors declare that the research was conducted in the absence of any commercial or financial relationships that could be construed as a potential conflict of interest.

## Publisher’s note

All claims expressed in this article are solely those of the authors and do not necessarily represent those of their affiliated organizations, or those of the publisher, the editors and the reviewers. Any product that may be evaluated in this article, or claim that may be made by its manufacturer, is not guaranteed or endorsed by the publisher.

## References

[ref2] Ajzen I. (1991). The theory of planned behavior. Organ. Behav. Hum. Decis. Process. 50, 179–211. doi: 10.1016/0749-5978(91)90020-T

[ref3] American Psychiatric Association. (2013). Diagnostic and statistical manual of mental disorders: DSM-5. 5th. Washington, D.C.: American Psychiatric Publishing.

[ref4] Araiba S. Čolić M. (2022). Preliminary practice recommendations for telehealth direct applied behavior analysis services with children with autism. J. Behav. Educ. 1-35, 1–35. doi: 10.1007/s10864-022-09473-6, PMID: 35464786 PMC9013273

[ref5] Arim R. G. Miller A. R. Guèvremont A. Lach L. M. Brehaut J. C. Kohen D. E. (2017). Children with neurodevelopmental disorders and disabilities: a population-based study of healthcare service utilization using administrative data. Dev. Med. Child Neurol. 59, 1284–1290. doi: 10.1111/dmcn.13557, PMID: 28905997

[ref6] Ashburner J. Vickerstaff S. Beetge J. Copley J. (2016). Remote versus face-to-face delivery of early intervention programs for children with autism spectrum disorders: perceptions of rural families and service providers. RASD 23, 1–14. doi: 10.1016/j.rasd.2015.11.011

[ref7] Awasthi S. Aravamudhan S. Jagdish A. Joshi B. Mukherjee P. Kalkivaya R.. (2021). Transitioning ABA services from in clinic to telehealth: case study of an Indian organization’s response to COVID-19 lockdown. Behav. Anal. Pract. 14, 893–912. doi: 10.1007/s40617-021-00600-9, PMID: 34394851 PMC8356690

[ref8] Barretto A. Wacker D. P. Harding J. Lee J. Berg W. K. (2006). Using telemedicine to conduct behavioral assessments. JABA 39, 333–340. doi: 10.1901/jaba.2006.173-04, PMID: 17020213 PMC1702392

[ref9] Batton B. Kaplan R. Ellis K. Schmidt C. Nudelman E. (2022). Telehealth training in principles of applied behavior analysis for caregivers of young children with autism spectrum disorders during the COVID-19 pandemic. ETC 45, 299–303. doi: 10.1007/s43494-022-00081-7, PMID: 35936026 PMC9345387

[ref10] Blackman A. L. Jimenez-Gomez C. Shvarts S. (2020). Comparison of the efficacy of online versus in-vivo behavior analytic training for parents of children with autism spectrum disorder. Behav. Anal. Res. Pract. 20, 13–23. doi: 10.1037/bar0000163

[ref11] Camden C. Silva M. (2021). Pediatric teleheath: opportunities created by the COVID-19 and suggestions to sustain its use to support families of children with disabilities. Phys. Occup. Ther. Pediatr. 41, 1–17. doi: 10.1080/01942638.2020.1825032, PMID: 33023352

[ref12] Campbell J. Theodoros D. Russell T. Gillespie N. Hartley N. (2019). Client, provider and community referrer perceptions of telehealth for the delivery of rural paediatric allied health services. AJRH 27, 419–426. doi: 10.1111/ajr.12519, PMID: 31571313

[ref14] Clarke V. Braun V. Hayfield N. (2015). “Thematic analysis” in Qualitative psychology: A practical guide to research methods. ed. Smith J. A. (London: Sage Publications Ltd.), 222–248.

[ref15] Colombo R. A. Wallace M. Taylor R. (2020). An essential service decision model for ABA providers during crisis. Behav. Anal. Pract. 13, 306–311. doi: 10.1007/s40617-020-00432-z, PMID: 32637293 PMC7243734

[ref16] Cox D. J. Plavnick J. B. Brodhead M. T. (2020). A proposed process for risk mitigation during the COVID-19 pandemic. Behav. Anal. Pract. 13, 299–305. doi: 10.1007/s40617-020-00430-1, PMID: 32328220 PMC7178923

[ref17] Degli Espinosa F. Metko A. Raimondi M. Impenna M. Scognamiglio E. (2020). A model of support for families of children with autism living in the COVID-19 lockdown: lessons from Italy. Behav. Anal. Pract. 13, 550–558. doi: 10.1007/s40617-020-00438-7, PMID: 32837696 PMC7266382

[ref18] Dueñas A. D. D'Agostino S. R. (2022). Experiences of service providers in the expedited delivery of ABA therapy via telehealth during the COVID-19 pandemic: reflections and considerations for the future. Behav. Anal. Res. Pract. 22, 265–282. doi: 10.1037/bar0000251

[ref19] Ferguson J. Craig E. A. Dounavi K. (2019). Telehealth as a model for providing behaviour analytic interventions to individuals with autism spectrum disorder: a systematic review. J. Autism Dev. Disord. 49, 582–616. doi: 10.1007/s10803-018-3724-5, PMID: 30155578 PMC6373531

[ref20] Ferguson J. Dounavi K. Craig E. A. (2022). The impact of a telehealth platform on ABA-based parent training targeting social communication in children with autism spectrum disorder. J. Dev. Phys. Disabil. 34, 1089–1120. doi: 10.1007/s10882-022-09839-8, PMID: 35370389 PMC8961090

[ref21] Foxx R. M. (2008). Applied behavior analysis treatment of autism: the state of the art. Child Adolesc. Psychiatr. Clin. N. Am. 17, 821–834. doi: 10.1016/j.chc.2008.06.007, PMID: 18775372

[ref23] Gerow S. Kirkpatrick M. McGinnis K. Sulak T. N. Davis T. N. Fritz S. (2022). Evaluation of a telehealth ABA program for caregivers of children with ASD. Behav. Modif. 47, 349–379. doi: 10.1177/01454455221130001, PMID: 36317793

[ref25] Gros D. F. Morland L. A. Greene C. J. Acierno R. Strachan M. Egede L. E.. (2013). Delivery of evidence-based psychotherapy via video telehealth. J. Psychopathol. Behav. Assess. 35, 506–521. doi: 10.1007/s10862-013-9363-4

[ref26] Hao Y. Zhang S. Conner A. Lee N. Y. (2021). The evolution of telepractice use during the COVID-19 pandemic: perspectives of pediatric speech-language pathologists. IJERPH 18:12197. doi: 10.3390/ijerph182212197, PMID: 34831952 PMC8620697

[ref27] Hoffmann A. N. Bogoev B. K. Sellers T. P. (2019). Using telehealth and expert coaching to support early childhood special education parent-implemented assessment and intervention procedures. RSEQ 38, 95–106. doi: 10.1177/8756870519844162

[ref28] Kryszak E. M. Albright C. M. Fell L. A. Butter E. M. Kuhlthau K. A. (2022). Clinician perspectives on telehealth assessment of autism spectrum disorder during the COVID-19 pandemic. J. Autism Dev. Disord. 52, 5083–5098. doi: 10.1007/s10803-022-05435-z, PMID: 35103899 PMC8804366

[ref29] Lee J. F. Schieltz K. M. Suess A. N. Wacker D. P. Romani P. W. Lindgren S. D.. (2015). Guidelines for developing telehealth services and troubleshooting problems with telehealth technology when coaching parents to conduct functional analyses and functional communication training in their homes. Behav. Anal. Pract. 8, 190–200. doi: 10.1007/s40617-014-0031-2, PMID: 27703918 PMC5048262

[ref30] Lerman D. C. O’Brien M. J. Neely L. Call N. A. Tsami L. Schieltz K. M.. (2020). Remote coaching of caregivers via telehealth: challenges and potential solutions. J. Behav. Educ. 29, 195–221. doi: 10.1007/s10864-020-09378-2, PMID: 36093285 PMC9455948

[ref31] Lindgren S. Wacker D. Schieltz K. Suess A. Pelzel K. Kopelman T.. (2020). A randomized controlled trial of functional communication training via telehealth for young children with autism spectrum disorder. J. Autism Dev. Disord. 50, 4449–4462. doi: 10.1007/s10803-020-04451-1, PMID: 32300910 PMC7572463

[ref32] Lindgren S. Wacker D. Suess A. Schieltz K. Pelzel K. Kopelman T.. (2016). Telehealth and autism: treating challenging behavior at lower cost. Pediatrics 137, S167–S175. doi: 10.1542/peds.2015-2851O, PMID: 26908472 PMC4727312

[ref34] Palinkas L. A. Aarons G. A. Horwitz S. Chamberlain P. Hurlburt M. Landsverk J. (2011). Mixed method designs in implementation research. Admin. Pol. Ment. Health 38, 44–53. doi: 10.1007/s10488-010-0314-z, PMID: 20967495 PMC3025112

[ref35] Peterson K. M. Piazza C. C. Luczynski K. C. Fisher W. W. (2017). Virtual-care delivery of applied-behavior-analysis services to children with autism spectrum disorder and related conditions. Behav. Anal. Res. Pract. 17, 286–297. doi: 10.1037/bar0000030

[ref36] Pew Research Center. (2021). Mobile fact sheet. Available at: https://www.pewresearch.org/internet/fact-sheet/mobile/ (accessed February 8, 2023).

[ref37] Pollard J. S. LeBlanc L. A. Griffin C. A. Baker J. M. (2021). The effects of transition to technician-delivered telehealth ABA treatment during the COVID-19 crisis: a preliminary analysis. JABA 54, 87–102. doi: 10.1002/jaba.803, PMID: 33369729 PMC7898711

[ref38] Proctor E. Silmere H. Raghavan R. Hovmand P. Aarons G. Bunger A.. (2011). Outcomes for implementation research: conceptual distinctions, measurement challenges, and research agenda. Admin. Pol. Ment. Health 38, 65–76. doi: 10.1007/s10488-010-0319-7, PMID: 20957426 PMC3068522

[ref39] Rodriguez K. A. (2020). Maintaining treatment integrity in the face of crisis: a treatment selection model for transitioning direct ABA services to telehealth. Behav. Anal. Pract. 13, 291–298. doi: 10.1007/s40617-020-00429-8, PMID: 32426099 PMC7232927

[ref40] Romani P. W. Schieltz K. M. (2017). Ethical considerations when delivering behavior analytic services for problem behavior via telehealth. Behav. Anal. Res. Pract. 17, 312–324. doi: 10.1037/bar0000074, PMID: 29333486 PMC5764537

[ref41] Rooks-Ellis D. L. Howorth S. K. Boulette S. Kunze M. Sulinski E. (2020). Effects of a parent training using telehealth: equity and access to early intervention for rural families. JCES 1, 141–166. doi: 10.37291/2717638X.20201242

[ref42] Schieltz K. M. Wacker D. P. (2020). Functional assessment and function-based treatment delivered via telehealth: a brief summary. JABA 53, 1242–1258. doi: 10.1002/jaba.742, PMID: 32643811 PMC7361834

[ref43] Southey S. J. Stoddart K. P. (2021). Clinical intervention with autistic adolescents and adults during the first two months of the COVID-19 pandemic: experiences of clinicians and their clients. Int. Soc. Work. 64, 777–782. doi: 10.1177/00208728211012462

[ref44] Spain D. Mason D. Capp S. J. Stoppelbein L. White S. W. Happé F. (2021). “This may be a really good opportunity to make the world a more autism friendly place”: professionals’ perspectives on the effects of COVID-19 on autistic individuals. RASD 83:101747. doi: 10.1016/j.rasd.2021.101747 PMC9760644 36570074

[ref45] Sutherland R. Trembath D. Hodge M. A. Rose V. Roberts J. (2019). Telehealth and autism: are telehealth language assessments reliable and feasible for children with autism? IJLCD 54, 281–291. doi: 10.1111/1460-6984.12440, PMID: 30565791

[ref46] Tabak R. G. Khoong E. C. Chambers D. A. Brownson R. C. (2012). Bridging research and practice: models for dissemination and implementation research. Am. J. Prev. Med. 43, 337–350. doi: 10.1016/j.amepre.2012.05.024, PMID: 22898128 PMC3592983

[ref47] Tomlinson S. R. Gore N. McGill P. (2018). Training individuals to implement applied behavior analytic procedures via telehealth: a systematic review of the literature. J. Behav. Educ. 27, 172–222. doi: 10.1007/s10864-018-9292-0

[ref48] Unholz-Bowden E. McComas J. J. McMaster K. L. Girtler S. N. Kolb R. L. Shipchandler A. (2020). Caregiver training via telehealth on behavioral procedures: a systematic review. J. Behav. Educ. 29, 246–281. doi: 10.1007/s10864-020-09381-7 PMC10479951 37670908

[ref49] Weiner B. J. Lewis C. C. Stanick C. Powell B. J. Dorsey C. N. Clary A. S.. (2017). Psychometric assessment of three newly developed implementation outcome measures. Implement. Sci. 12, 108–112. doi: 10.1186/s13012-017-0635-3, PMID: 28851459 PMC5576104

[ref50] White S. W. Stoppelbein L. Scott H. Spain D. (2021). It took a pandemic: perspectives on impact, stress, and telehealth from caregivers of people with autism. Res. Dev. Disabil. 113:103938. doi: 10.1016/j.ridd.2021.103938, PMID: 33730684 PMC9758058

